# Modeling and Characterization of Multilayer Piezoelectric Stacks via Dynamic Stiffness Method

**DOI:** 10.3390/mi16010020

**Published:** 2024-12-26

**Authors:** Wenxiang Ding, Zhaofeng Liang, Wei Zhao, Hongmei Zhong, Dan Chen, Maxime Bavencoffe, Marc Lethiecq

**Affiliations:** 1 School of Mechanical and Electrical Engineering, Shenzhen Polytechnic University, No. 7098, Liuxian Avenue, Shenzhen 518055, China; zhaowei@szpu.edu.cn (W.Z.); hmzhong@szpu.edu.cn (H.Z.); 2 Institute of Ultrasonic Technology, Shenzhen Polytechnic University, No. 7098, Liuxian Avenue, Shenzhen 518055, China; danchen@szpu.edu.cn; 3 GREMAN UMR 7347 CNRS, Université de Tours, INSA Centre Val de Loire, 3 Rue de la Chocolaterie, 41000 Blois, France; maxime.bavencoffe@insa-cvl.fr (M.B.); marc.lethiecq@insa-cvl.fr (M.L.)

**Keywords:** multilayer piezoelectric stack, dynamic stiffness method, electrical impedance, mode shape, finite element method

## Abstract

Multilayer piezoelectric stacks, which are multiple layers of piezoelectric materials placed on top of each other, are widely used to achieve precise linear movement and high-force generation. In this paper, a dynamic stiffness (DS) method for the dynamic vibration analysis of multilayer piezoelectric stacks is presented. First, the general solutions for all physical quantities of the three vibration contributions (i.e., pure vibration, symmetrically coupled vibration, and anti-symmetrically coupled vibration) are derived from the governing equations of motion. Then, the DS matrices of each layer of the piezoelectric stack are obtained, and they are assembled to form a global DS matrix. The electrical impedances and the mode shapes of a piezoelectric stack consisting of two piezoelectric disks connected in series and in parallel are calculated using our method as well as by the finite element method. The comparison shows good agreement. Finally, the effect of the number of layers on the dynamic responses of piezoelectric stacks is investigated. The DS method developed here provides an efficient and accurate analytical tool for the parametric and optimization analysis of the coupled vibrations of multilayer piezoelectric structures.

## 1. Introduction

Multilayer piezoelectric stacks are widely used as sensors and actuators in high-precision positioners, energy harvesters, electroacoustic equipment, etc [1,2,3]. In the design of such sensors or actuators, the effect of the geometry, the material properties, and the boundary conditions on performance should be investigated. Therefore, an efficient and accurate tool to simulate the electromechanical behavior of the devices is required.

Multilayer piezoelectric stacks are made by stacking multiple layers of piezoelectric resonators on top of each other. Although the vibration characteristics of a single-layer piezoelectric resonator are revealed by three sets of equations—algebraic, divergence, and gradient equations [4]—it is still difficult to obtain an exact analytical solution, even for a simple geometry. If the piezoelectric resonator is long or thin, such as rods and disks, the dominant vibration can be simplified to one-dimensional (1D) motion along the length or thickness of the structure. Their dynamic characteristics can then be described by Mason [5], Krimholtz et al. (KLM) [6], or the Van Dyke [7] circuit models. These models are derived under simplified state of stress assumptions and are only applicable for resonators that approximately conform to shapes with one “pure” vibration mode.

For the piezoelectric resonators with finite dimensions, the longitudinal and transverse vibration modes are inevitably coupled together, which can be described by a system of coupled partial differential equations (PDEs). The numerical methods used for coupled vibration analysis can be categorized into two groups: the finite element (FE) method and the approximation method. Several typical FE studies on the vibration characteristics of piezoelectric disks with different diameter-to-thickness ratios can be found in [8,9,10]. The PDEs are solved approximately based on the variational method, and the frequency-independent low-order polynomial shape functions are used to represent field variables. However, the FE method becomes impracticable when the vibration analysis is performed in the high-frequency ranges. The FE model must be discretized small enough to capture the structural deformations, resulting in excessive degrees of freedom and huge computational costs. This drawback becomes more prominent for optimization design and parametric studies.

Based on the assumption that the coordinate axes are pure vibration mode propagation directions, a three-dimensional (3D) approximation approach was proposed by Brissaud [11]. The displacements in the radial and thickness directions are expressed by Bessel’s equation of the first order and sine function, respectively. A more generalized form was developed by Iula et al. [12] and Lin [13] using two orthogonal functions, i.e., sine and cosine functions, as the solutions. However, the vibrations are weakly coupled in this kind of approach because the displacements depend only on one axis, and the electric field in the non-polarized direction is assumed to be zero, which, in fact, is not. The coupling between the thickness extensional and the other vibration modes of elastic plates was initially studied by Mindlin et al. [14,15]. The 3D equations of elasticity were converted to an infinite series of two-dimensional (2D) equations by expanding the displacements in an infinite series of powers of the thickness coordinate of the plate and integrating through the thickness. The theory was later extended to piezoelectric plates by Tiersten [16] and Lee et al. [17,18]. Although the vibrations are naturally coupled through the expressions of bivariate functions, the boundary conditions are satisfied in a weak integral form. Recently, a modified higher-order shear deformation plate theory was proposed to describe the vibration behavior of thick piezoelectric disks [19]. The deformation of the piezoelectric disk under electric actuation can be obtained without the need to solve the electric potential field. An accurate approximation approach based on the superposition method was proposed by some of the co-authors of this paper [20,21]. The vibration is decomposed into two building blocks: vibrations in the radial and thickness directions. The PDEs and boundary conditions are satisfied exactly in the form of Fourier and Fourier–Bessel series expansion. However, it can only be used for free or symmetric loads, and the form of the series expansion is not optimal.

For multilayer piezoelectric stacks, a few attempts have been made to parametrically model piezoelectric stacks over the past decades. There is no doubt that 1D circuit models can be extended to multilayer structures [22,23]. A non-linear lumped-parameter model of a piezoelectric stack actuator was developed by Goldfard et al. [24]. The mechanical and electrical sides are modeled by a lumped mass-spring-damper system and a lumped capacitance, respectively. Three- and five-port equivalent circuits, which offer an explicit representation of the electromechanical behaviors of multilayer piezoelectric stacks, were proposed by Ha et al. [1] and Zhang et al. [2]. Recently, a modified first-order plate theory of laminated piezoelectric plates was proposed by Lian et al. [25]. An electromechanical dynamic stiffness matrix in Taylor’s series was developed by Ling et al. [26] for both the kinetostatic and dynamic analysis of micro/nano motion actuators. However, all the aforementioned works assumed a linear distribution of electric potential in the thickness direction and focused mainly on the *d*_33_ vibration mode.

In the present work, a general DS method is presented for the dynamic response analysis of multilayer piezoelectric stacks of arbitrary dimensions. The DS matrix linking the generalized force and the generalized displacement of a single-layer piezoelectric ceramic disk has been explicitly derived for the first time. The global DS matrix of piezoelectric stacks with arbitrary boundary conditions is constructed using the same assemblage procedure as in the FE method. By imposing boundary conditions, dynamic responses can be obtained.

## 2. Equation of Motion

### 2.1. Basic Coupled Field Equations

**Figure 1:**
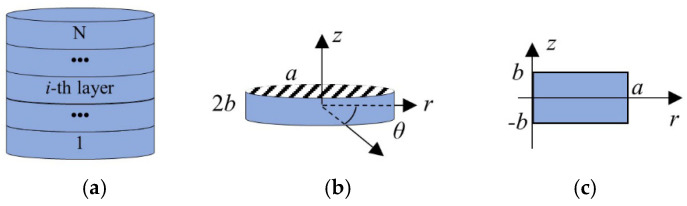
Schematic representation of a multilayer piezoelectric stack. (**a**) Several perfectly bonded and electrically connected piezoelectric elements; (**b**) three-dimensional (3D) model of a single-layer element; (**c**) two-dimensional (2D) axisymmetric model of a single-layer element.

Piezoelectric materials of 6 mm hexagonal crystal symmetry with five elastic, three piezoelectric, and two dielectric constants are considered here. Figure 1a shows a multilayer piezoelectric stack composed of *N* perfectly bonded and electrically connected layers. It is assumed that each layer is of the same material and size. The cylindrical coordinate system (*r*, *θ*, *z*) and geometric dimensions of the *i*-th single layer with a radius *a* and a thickness 2*b* are shown in Figure 1b. The piezoelectric element is poled in the thickness direction (parallel to the *z*-axis). Due to its axisymmetric structure, the 3D vibration problem of a single-layer element can be reduced to a 2D axisymmetric one, as shown in Figure 1c.

Based on the linear theory of piezoelectricity, the elasticity equations of motion and Maxwell’s equation of the 2D axisymmetric model are

(1) $$\begin{array}{cc} \begin{array}{l} T_{\alpha ,i}=\rho {\ddot{u}}_{j}\\ D_{i,i}=0 \end{array} & i,j=1,2,3\;\mathrm{and}\;\alpha =1,\ldots ,6 \end{array}$$

where *T_α_* and *D_i_* are elastic stress and electric displacement, *ρ* is the density, *u_j_* is the mechanical displacement, and dots above the variables denote the time derivative.

The linear constitutive equations are given by [27]

(2) $$\begin{array}{cc} \begin{array}{l} T_{\alpha }=c_{\alpha \beta }^{E}S_{\beta }-e_{i\alpha }E_{i}\\ D_{i}=e_{i\alpha }S_{\alpha }+\varepsilon_{ij}^{S}E_{j} \end{array} & \beta =1,\ldots ,6 \end{array}$$

where *S_β_* and *E_i_* are elastic strain and electric field. $c_{\alpha \beta }^{E}$ are the elastic stiffness constants under constant electric field, $e_{i\alpha }$ are the piezoelectric constants, and $\varepsilon_{ij}^{S}$ are the dielectric constants under constant strain. If the polarization direction is positive along the *z*-axis, the piezoelectric constants $e_{i\alpha }=e_{i\alpha }$, and otherwise, $e_{i\alpha }=-e_{i\alpha }$.

The definition equations for mechanical stress/strain and electric field/potential are

(3) $$\begin{array}{l} S_{ij}=\frac{1}{2}\left(u_{i,j}+u_{j,i}\right)\\ E_{i}=-\varphi_{,i} \end{array}$$

where *φ* is the electric potential, and the commas in subscripts denote the coordinate partial derivative.

For the 2D axisymmetric model in Figure 1c, the interaction between *θ*-axis and *rz*-plane can be omitted. Substituting Equations (2) and (3) into Equation (1), the vibration equations can be further simplified to a set of coupled field equations for mechanical displacement *u*_1_, *u*_3_ and electric potential *φ* as

(4) $$\begin{array}{l} c_{11}^{E}\nabla^{2}u_{1}+\left(c_{13}^{E}+c_{55}^{E}\right)u_{3,13}+c_{55}^{E}u_{1}{,}_{33}+\left(e_{31}+e_{15}\right)\varphi_{,13}=\rho {\ddot{u}}_{1}\\ c_{33}^{E}u_{3,33}+\left(c_{13}^{E}+c_{55}^{E}\right)\Delta u_{1}{,}_{3}+c_{55}^{E}\Delta u_{3,1}+e_{33}\varphi_{,33}+e_{15}\Delta \varphi_{,1}=\rho {\ddot{u}}_{3}\\ e_{15}\Delta u_{3,1}+\left(e_{15}+e_{31}\right)\Delta u_{1}{,}_{3}+e_{33}u_{3,33}-\varepsilon_{11}^{S}\Delta \varphi_{,1}-\varepsilon_{33}^{S}\varphi_{,33}=0 \end{array}$$

where *u*_1_, *u*_3_ are the displacements along the *r* and *z* axes, and the operators ∆ and ∇^2^ are defined as

(5) $$\Delta =\frac{\partial }{\partial r}+\frac{1}{r},\;\nabla^{2}=\frac{\partial }{\partial r}\Delta =\frac{\partial^{2}}{\partial r^{2}}+\frac{1}{r}\frac{\partial }{\partial r}-\frac{1}{r^{2}}$$

### 2.2. General Solutions

Finding suitable admissible functions for the three primary variables *u*_1_, *u*_3_, *φ* is essential for solving coupled vibration problems. The admissible functions are required to be independent, to be differentiable, to satisfy the coupled field Equation (4), and to satisfy the boundary conditions presented later in Section 3.1.2. This is not an easy task, and various forms have been provided by Mindlin and his students [14,18,28], where the primary variables are expressed as infinite power or trigonometric series expansions in thickness coordinates. As an extension of the previous studies [29,30,31], general solutions of the three primary variables are divided into three contributions: pure (*p*) vibration modes, symmetrically coupled (*sc*) vibration modes, and anti-symmetrically coupled (*ac*) vibration modes as

(6) $$\begin{array}{l} u_{1}=u_{1}^{p}+u_{1}^{sc}+u_{1}^{ac}\\ u_{3}=u_{3}^{p}+u_{3}^{sc}+u_{3}^{ac}\\ \varphi =\varphi^{p}+\varphi^{sc}+\varphi^{ac} \end{array}$$

#### 2.2.1. Pure Vibration Modes

The components of pure vibration modes can be represented by

(7) $$\begin{array}{l} u_{1}^{p}=A_{5}J_{1}\left(k_{2}r\right)\\ u_{3}^{p}=A_{1}\sin\left(k_{1}z\right)+A_{2}\cos\left(k_{1}z\right)\\ \varphi^{p}=\gamma_{1}\left[A_{1}\sin\left(k_{1}z\right)+A_{2}\cos\left(k_{1}z\right)\right]+A_{3}z+A_{4} \end{array}$$

where $\gamma_{1}=e_{33}/\varepsilon_{33}^{S}$, *A_i_* (*i* = 1,2,…,5) are the unknown coefficients, *J*_1_ is the Bessel function of the first kind of order one, and

(8) $$k_{1}=\sqrt{\frac{\rho }{c_{33}^{D}}}\omega ,\;k_{2}=\sqrt{\frac{\rho }{c_{11}^{E}}}\omega$$

are the wavenumber in the *z* and *r* directions, respectively. $c_{33}^{D}$ is the elastic stiffness constant under constant electric displacement, and *ω* is the angular frequency.

Insertion of Equations (7) and (3) into Equation (2) leads to the solutions of stresses and electric displacements.

(9) $$\begin{array}{l} T_{3}^{p}{=\beta }_{1}\left(\cos\left(k_{1}z\right)A_{1}-\sin\left(k_{1}z\right)A_{2}\right)+\gamma_{2}A_{3}+\beta_{2}A_{5}J_{0}\left(k_{2}r\right)\\ T_{1}^{p}=\beta_{3}\left(\cos\left(k_{1}z\right)A_{1}-\sin\left(k_{1}z\right)A_{2}\right)+\gamma_{3}A_{3}+\left(\beta_{4}J_{0}\left(k_{2}r\right)+\frac{\gamma_{5}}{r}J_{1}\left(k_{2}r\right)\right)A_{5}\\ D_{3}^{p}=-\gamma_{4}A_{3}+\beta_{5}J_{0}\left(k_{2}r\right)A_{5}\\ T_{5}^{p}=D_{1}^{p}=0 \end{array}$$

where $\beta_{1}=c_{33}^{D}k_{1}$, $\beta_{2}=c_{13}^{E}k_{2}$, $\beta_{3}=c_{13}^{D}k_{1}$, $\beta_{4}=c_{11}^{E}k_{2}$, $\gamma_{2}=e_{33}$, $\gamma_{3}=e_{31}$, $\gamma_{4}=\varepsilon_{33}^{S}$, and $\gamma_{5}=c_{12}^{E}-c_{11}^{E}$.

We note that the expressions of the mechanical displacements $u_{1}^{p}$ and $u_{3}^{p}$ in Equation (7) are the same as those in [12]; however, the expression of the electric field $E_{3}$ therein (shown in Equation (10)) is not derived properly. To deal with the dependence of *r*, *h*_31_ = 0 must be imposed, or an integral along *r*-axis should be implemented. In addition, the electric filed $E_{3}$ in most works related to the piezoelectric stacks [1,2] is assumed to be linear in the thickness direction, i.e., $E_{3}=V/2b$, which, in fact, violates Maxwell’s Equation (1).

(10) $$E_{3}=\frac{h_{31}k_{1}}{j\omega }\frac{J_{0}\left(k_{1}r\right)}{J_{0}\left(k_{1}a\right)}u_{1}+\frac{h_{33}k_{3}}{j\omega }\left[\frac{u_{2}+u_{3}}{2\sin\left(k_{3}b\right)}\cos\left(k_{3}z\right)\right.\left.-\frac{u_{2}-u_{3}}{2\cos\left(k_{3}b\right)}\sin\left(k_{3}z\right)\right]+\beta_{33}^{S}D_{3}$$

By virtue of Equation (3), the electric field here can be elegantly expressed as

(11) $$E_{3}=-\frac{\partial \varphi^{p}}{\partial z}=-k_{1}\gamma_{1}\left[A_{1}\cos\left(k_{1}z\right)-A_{2}\sin\left(k_{1}z\right)\right]-A_{3}$$

#### 2.2.2. Symmetrically Coupled Vibration Modes

The components of symmetrically coupled vibration modes are represented by

(12) $$\begin{array}{l} u_{1}^{sc}=\sum_{me=1}^{+\infty }H_{0}\left(k_{mei}z\right)J_{1}\left(k_{me}r\right)+\sum_{ne=1}^{+\infty }G_{0}\left(k_{nei}r\right)\cos\left(k_{ne}z\right)\\ u_{3}^{sc}=\sum_{me=1}^{+\infty }H_{1}\left(k_{mei}z\right)J_{0}\left(k_{me}r\right)+\sum_{ne=1}^{+\infty }G_{1}\left(k_{nei}r\right)\sin\left(k_{ne}z\right)\\ \varphi^{sc}=\sum_{me=1}^{+\infty }H_{2}\left(k_{mei}z\right)J_{0}\left(k_{me}r\right)+\sum_{ne=1}^{+\infty }G_{2}\left(k_{nei}r\right)\sin\left(k_{ne}z\right) \end{array}$$

where *J*_0_ is the Bessel function of the first kind of order zero. The wavenumber in the radial direction, *k_me_*, is the *me*-th (*me* > 0) root of *J*_1_(*k_me_a*) = 0. The wavenumber in the thickness direction is defined as *k_ne_* = *ne* × π/*b*, (*ne* = 1,2,…,+ꝏ). The functions $H_{j}$ and $G_{j}$ (*j* = 0,1,2) are given by

(13) $$\begin{array}{ll} H_{0}\left(k_{mei}z\right)=\sum_{i=1}^{3}A_{mei}\cos\left(k_{mei}z\right), & G_{0}\left(k_{nei}r\right)=\sum_{i=1}^{3}A_{nei}J_{1}\left(k_{nei}r\right)\\ H_{1}\left(k_{mei}z\right)=\sum_{i=1}^{3}\alpha_{1}^{mei}A_{mei}\sin\left(k_{mei}z\right), & G_{1}\left(k_{nei}r\right)=\sum_{i=1}^{3}\alpha_{1}^{nei}A_{nei}J_{0}\left(k_{nei}r\right)\\ H_{2}\left(k_{mei}z\right)=\sum_{i=1}^{3}\alpha_{2}^{mei}A_{mei}\sin\left(k_{mei}z\right), & G_{2}\left(k_{nei}r\right)=\sum_{i=1}^{3}\alpha_{2}^{nei}A_{nei}J_{0}\left(k_{nei}r\right) \end{array}$$

where definitions of the coefficients $\alpha_{1}^{mei}$, $\alpha_{2}^{mei}$, $\alpha_{1}^{nei}$, and $\alpha_{2}^{nei}$ are detailed in Appendix A.

Insertion of Equations (12) and (3) into Equation (2) leads to the solutions of stresses and electric displacements.

(14) $$\begin{array}{cc} T_{3}^{sc} & =\sum_{me=1}^{+\infty }H_{3}\left(k_{mei}z\right)J_{0}\left(k_{me}r\right)+\sum_{ne=1}^{+\infty }G_{3}\left(k_{nei}r\right)\cos\left(k_{ne}z\right)\\ T_{1}^{sc} & =\sum_{me=1}^{+\infty }H_{4}\left(k_{mei}z\right)J_{0}\left(k_{me}r\right)+\sum_{ne=1}^{+\infty }G_{4}\left(k_{nei}r\right)\cos\left(k_{ne}z\right)\\ & +\sum_{me=1}^{+\infty }{\bar{H}}_{4}\left(k_{mei}z\right)\frac{J_{1}\left(k_{me}r\right)}{r}+\sum_{ne=1}^{+\infty }{\bar{G}}_{4}\left(k_{nei}r\right)\cos\left(k_{ne}z\right)\\ T_{5}^{sc} & =\sum_{me=1}^{+\infty }H_{5}\left(k_{mei}z\right)J_{1}\left(k_{me}r\right)+\sum_{ne=1}^{+\infty }G_{5}\left(k_{nei}r\right)\sin\left(k_{ne}z\right)\\ D_{3}^{sc} & =\sum_{me=1}^{+\infty }H_{6}\left(k_{mei}z\right)J_{0}\left(k_{me}r\right)+\sum_{ne=1}^{+\infty }G_{6}\left(k_{nei}r\right)\cos\left(k_{ne}z\right)\\ D_{1}^{sc} & =\sum_{me=1}^{+\infty }H_{7}\left(k_{mei}z\right)J_{1}\left(k_{me}r\right)+\sum_{ne=1}^{+\infty }G_{7}\left(k_{nei}r\right)\sin\left(k_{ne}z\right) \end{array}$$

where definitions of the functions ${\bar{H}}_{4}\left(k_{mei}z\right)$, ${\bar{G}}_{4}\left(k_{nei}r\right)$, $H_{j}\left(k_{mei}z\right)$ and $G_{j}\left(k_{nei}r\right)$ (*j* = 3,…,7) are also provided in Appendix A.

#### 2.2.3. Anti-Symmetrically Coupled Vibration Modes

The components of anti-symmetrically coupled vibration modes are represented by

(15) $$\begin{array}{l} u_{1}^{ac}=\sum_{me=1}^{+\infty }{H^{\prime }}_{0}\left(k_{mei}z\right)J_{1}\left(k_{me}r\right)+\sum_{no=1}^{+\infty }{G^{\prime }}_{0}\left(k_{noi}r\right)\sin\left(k_{no}z\right)\\ u_{3}^{ac}=\sum_{me=1}^{+\infty }{H^{\prime }}_{1}\left(k_{mei}z\right)J_{0}\left(k_{me}r\right)+\sum_{no=1}^{+\infty }{G^{\prime }}_{1}\left(k_{noi}r\right)\cos\left(k_{no}z\right)\\ \varphi^{ac}=\sum_{me=1}^{+\infty }{H^{\prime }}_{2}\left(k_{mei}z\right)J_{0}\left(k_{me}r\right)+\sum_{no=1}^{+\infty }{G^{\prime }}_{2}\left(k_{noi}r\right)\cos\left(k_{no}z\right) \end{array}$$

where the definition of the wavenumber in radial direction *k_me_* is the same as Equation (12). The wavenumber in the thickness direction is defined as *k_no_* = (*no* − 1/2) × π/*b*, (*no* = 1,2,…,+ꝏ). The functions ${H^{\prime }}_{j}\left(k_{mei}z\right)$ and ${G^{\prime }}_{j}\left(k_{noi}r\right)$ (*j* = 0,1,2) are given by

(16) $$\begin{array}{ll} {H^{\prime }}_{0}\left(k_{mei}z\right)=\sum_{i=1}^{3}A_{mei}\sin\left(k_{mei}z\right), & {G^{\prime }}_{0}\left(k_{noi}r\right)=\sum_{i=1}^{3}A_{noi}J_{1}\left(k_{noi}r\right)\\ {H^{\prime }}_{1}\left(k_{mei}z\right)=-\sum_{i=1}^{3}\alpha_{1}^{mei}A_{mei}\cos\left(k_{mei}z\right), & {G^{\prime }}_{1}\left(k_{noi}r\right)=-\sum_{i=1}^{3}\alpha_{1}^{noi}A_{noi}J_{0}\left(k_{noi}r\right)\\ {H^{\prime }}_{2}\left(k_{mei}z\right)=-\sum_{i=1}^{3}\alpha_{2}^{mei}A_{mei}\cos\left(k_{mei}z\right), & {G^{\prime }}_{2}\left(k_{noi}r\right)=-\sum_{i=1}^{3}\alpha_{2}^{noi}A_{noi}J_{0}\left(k_{noi}r\right) \end{array}$$

where definitions of the coefficients $\alpha_{1}^{noi}$, and $\alpha_{2}^{noi}$ can be easily obtained by changing $k_{ne}$ to $k_{no}$ in Appendix A.

Insertion of Equations (15) and (3) into Equation (2) leads to the solutions of stresses and electric displacements.

(17) $$\begin{array}{cc} T_{3}^{ac} & =\sum_{me=1}^{+\infty }{H^{\prime }}_{3}\left(k_{mei}z\right)J_{0}\left(k_{me}r\right)+\sum_{no=1}^{+\infty }{G^{\prime }}_{3}\left(k_{noi}r\right)\sin\left(k_{no}z\right)\\ T_{1}^{ac} & =\sum_{me=1}^{+\infty }{H^{\prime }}_{4}\left(k_{mei}z\right)J_{0}\left(k_{me}r\right)+\sum_{no=1}^{+\infty }{G^{\prime }}_{4}\left(k_{noi}r\right)\sin\left(k_{no}z\right)\\ & +\sum_{me=1}^{+\infty }{{\bar{H}}^{\prime }}_{4}\left(k_{mei}z\right)\frac{J_{1}\left(k_{me}r\right)}{r}+\sum_{ne=1}^{+\infty }{{\bar{G}}^{\prime }}_{4}\left(k_{noi}r\right)\sin\left(k_{no}z\right)\\ T_{5}^{ac} & =-\sum_{me=1}^{+\infty }{H^{\prime }}_{5}\left(k_{mei}z\right)J_{1}\left(k_{me}r\right)-\sum_{no=1}^{+\infty }{G^{\prime }}_{5}\left(k_{noi}r\right)\cos\left(k_{no}z\right)\\ D_{3}^{ac} & =\sum_{me=1}^{+\infty }{H^{\prime }}_{6}\left(k_{mei}z\right)J_{0}\left(k_{me}r\right)+\sum_{no=1}^{+\infty }{G^{\prime }}_{6}\left(k_{noi}r\right)\sin\left(k_{no}z\right)\\ D_{1}^{ac} & =-\sum_{me=1}^{+\infty }{H^{\prime }}_{7}\left(k_{mei}z\right)J_{1}\left(k_{me}r\right)-\sum_{no=1}^{+\infty }{G^{\prime }}_{7}\left(k_{noi}r\right)\cos\left(k_{no}z\right) \end{array}$$

where definitions of the functions ${{\bar{H}}^{\prime }}_{4}\left(k_{mei}z\right)$, ${{\bar{G}}^{\prime }}_{4}\left(k_{noi}r\right)$, ${H^{\prime }}_{j}\left(k_{mei}z\right)$, and ${G^{\prime }}_{j}\left(k_{noi}r\right)$ (*j* = 3,…,7) can also be easily obtained by changing $k_{ne}$ to $k_{no}$ and sine function to cosine function in Appendix A.

Different from the theory proposed by Mindlin and his students [14,18,28], the solutions of coupled vibrations here are expressed not only in terms of infinite trigonometric series expansions in the thickness *z* coordinate but also include infinite Bessel series expansions in the radial *r* coordinate. The boundary conditions can be satisfied exactly at every point of the boundary without integration.

## 3. Dynamic Stiffness Matrix and Boundary Conditions

The general solutions obtained previously will serve as the frequency-dependent dynamic shape functions to develop the DS matrix in this section. Then, based on the boundary conditions, the dynamic responses can be calculated.

### 3.1. DS Matrix of Single-Layer Piezoelectric Element

The generalized force vector **F***^ele^* and generalized displacement vector **U***^ele^* along the boundary *z* = ±*b* and *r* = *a* of the piezoceramic resonator in Figure 1c are defined as

(18) $$\begin{array}{ll} F^{ele} & ={\left[\begin{array}{lllllllll} F_{1} & F_{2} & F_{3} & F_{4} & F_{5} & F_{6} & F_{7} & F_{8} & F_{9} \end{array}\right]}^{T}\\ & ={\left[\begin{array}{lllllllll} T_{3}{|}_{z=b} & T_{5}{|}_{z=b} & \varphi {|}_{z=b} & T_{1}{|}_{r=a} & T_{5}{|}_{r=a} & D_{1}{|}_{r=a} & T_{3}{|}_{z=-b} & T_{5}{|}_{z=-b} & \varphi {|}_{z=-b} \end{array}\right]}^{T}\\ U^{ele} & ={\left[\begin{array}{ccccccccc} U_{1} & U_{2} & U_{3} & U_{4} & U_{5} & U_{6} & U_{7} & U_{8} & U_{9} \end{array}\right]}^{T}\\ & ={\left[\begin{array}{ccccccccc} u_{3}{|}_{z=b} & u_{1}{|}_{z=b} & D_{3}{|}_{z=b} & u_{1}{|}_{r=a} & u_{3}{|}_{r=a} & \varphi {|}_{r=a} & u_{3}{|}_{z=-b} & u_{1}{|}_{z=-b} & D_{3}{|}_{z=-b} \end{array}\right]}^{T} \end{array}$$

The generalized displacement and generalized force along the edge *z* = ±*b* are continuous functions of the variable *r*, and those along the edge *r* = *a* are continuous functions of the variable *z*. To deal with this spatial dependence, the Projection method [32] is adopted, and the generalized displacement and force are projected onto a set of the basis function *f* (*r*,*z*) as

(19) $${\tilde{F}}_{i}^{ele}\approx \sum_{mn=0}^{MN}\langle F_{i}^{ele},f_{i}\left(r,z\right)\rangle f_{i}\left(r,z\right),{\tilde{U}}_{i}^{ele}\approx \sum_{mn=0}^{MN}\langle U_{i}^{ele},f_{i}\left(r,z\right)\rangle f_{i}\left(r,z\right)$$

where the basis functions *f* (*r*,*z*) at the boundaries *z* = ±*b* are *J*_0_(*k_me_ r*) and *J*_1_(*k_me_ r*), the number of terms used for *k_me_* is *M*, that is, the symbol *mn* and *MN* represent *me* and *M,* respectively; the basis functions *f* (*r*,*z*) at the boundaries *r* = *a* are cos(*k_ne_ z*), sin(*k_ne_ z*), cos(*k_no_ z*), and sin(*k_no_ z*), and the number of terms used for *k_ne_* and *k_no_* is *N*, that is, the symbol *mn* and *MN* represent *ne/no* and *N,* respectively.

The projected force and displacement can be represented by

(20) $${\tilde{F}}^{ele}=P\left(\omega \right)A,\;\;\;\;{\tilde{U}}^{ele}=Q\left(\omega \right)A$$

where **P**(*ω*) and **Q**(*ω*) are 6 × (*M* + *N*) + 5 square matrices and the unknown coefficient **A** is given by

(21) $$A={\left[\begin{array}{cccccccc} A_{1} & A_{2} & A_{3} & A_{4} & A_{5} & A_{mei} & A_{nei} & A_{noi} \end{array}\right]}_{6\times \left(M+N\right)+5}^{T}$$

After eliminating the unknown coefficient in Equation (20), the DS matrix **K***^ele^*(*ω*) can be obtained.

(22) $${\tilde{F}}^{ele}=K^{ele}\left(\omega \right){\tilde{U}}^{ele},\;\;\;\;K^{ele}\left(\omega \right)=P\left(\omega \right)Q^{-1}\left(\omega \right)$$

#### 3.1.1. DS Matrix of Multilayer Piezoelectric Stack

**Figure 2:**
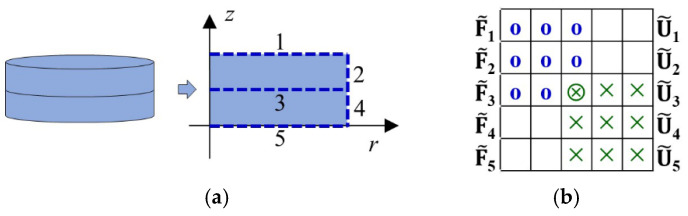
Assembly procedure of global dynamic stiffness (DS) matrix. (**a**) A multilayer piezoelectric stack consisting of two piezoelectric ceramic disks; (**b**) schematic diagram of assembly process.

Based on the above theory, a MATLAB® R2023a [33] program is developed for the free vibration analysis of multilayer piezoelectric stacks. The DS matrix of each individual piezoelectric element is assembled to obtain the global DS matrix of a multilayer piezoelectric stack. The assembly procedure is similar to that in the FE method, except the elements are connected along the boundary lines instead of nodes. The assembly procedure is demonstrated by an example of a piezoceramic stack in Figure 2. which consists of two piezoelectric ceramic disks connected along the boundary line-3 and ${\tilde{F}}_{i}$, ${\tilde{U}}_{i}$ $\left(i=1,\ldots ,5\right)$ represent the generalized forces and displacements along the boundary line-*i*.

The global dynamic stiffness matrix **K***^glo^*(*ω*) is given by

(23) $${\tilde{F}}^{glo}=K^{glo}\left(\omega \right){\tilde{U}}^{glo}$$

Assuming that the number of the terms *M* and *N* used for individual piezoelectric elements are the same, the size of the global DS matrix is

(24) $$N_{dof}=\left(3M+2\right)\times \left(n_{ele}+1\right)+\left(6N+1\right)\times n_{ele}$$

where $n_{ele}$ is the number of piezoelectric elements, (3*M* + 2) is the number of projections per vertical boundary lines (line-1, line-3, and line-5 in Figure 2a), and (6*N* + 1) is the number of projections per circumferential boundary lines (line-2 and line-4 in Figure 2a).

#### 3.1.2. Boundary and Continuity Conditions

Two multilayer piezoelectric stack arrangements are considered here: series-type and parallel-type. In series-type piezoelectric stacks, the adjacent piezoelectric elements have opposite poling directions; in parallel-type piezoelectric stacks, the poling directions are the same [34]. Taking a piezoelectric vibration energy harvester as an example, in series connectivity, higher voltage and low current are achieved, whereas higher current and low voltage are attained in parallel connection configuration [35]. The electric potentials of both types are applied to the bottom and top faces through thin electrodes. Therefore, the boundary conditions on the external surfaces can be written as

(25) $$\begin{array}{l} T_{1}^{i}{|}_{r=a}=T_{5}^{i}{|}_{r=a}=0,\;\;\;D_{1}^{i}{|}_{r=a}=0\;\left(i=1,\ldots ,N\right)\\ T_{3}^{N}{|}_{z=b}=T_{5}^{N}{|}_{z=b}=0,\;\;\;\varphi^{N}{|}_{z=b}=\varphi_{1}e^{i\omega t}\\ T_{3}^{1}{|}_{z=-b}=T_{5}^{1}{|}_{z=-b}=0,\;\;\;\varphi^{1}{|}_{z=-b}=\varphi_{2}e^{i\omega t} \end{array}$$

The continuity condition of the *i*-th layer at the internal interface *z* = ±*b* can be expressed as

(26) $$\begin{array}{l} \varphi^{i}{|}_{z=-b}=\varphi^{i-1}{|}_{z=b},D_{z}^{i}{|}_{z=-b}=D_{z}^{i-1}{|}_{z=b},u_{1}^{i}{|}_{z=-b}=u_{1}^{i-1}{|}_{z=b},\\ u_{3}^{i}{|}_{z=-b}=u_{3}^{i-1}{|}_{z=b},T_{3}^{i}{|}_{z=-b}=T_{3}^{i-1}{|}_{z=b},T_{5}^{i}{|}_{z=-b}=T_{5}^{i-1}{|}_{z=b}\\ \varphi^{i}{|}_{z=b}=\varphi^{i+1}{|}_{z=-b},D_{z}^{i}{|}_{z=b}=D_{z}^{i+1}{|}_{z=-b},u_{1}^{i}{|}_{z=b}=u_{1}^{i+1}{|}_{z=-b},\\ u_{3}^{i}{|}_{z=b}=u_{3}^{i+1}{|}_{z=-b},T_{3}^{i}{|}_{z=b}=T_{3}^{i+1}{|}_{z=-b},T_{5}^{i}{|}_{z=b}=T_{5}^{i+1}{|}_{z=-b} \end{array}$$

The dynamic responses of a multilayer piezoelectric stack in series-type or in parallel-type arrangements can be obtained by substituting boundary conditions into Equation (23).

## 4. Results and Discussion

To verify the validity of the DS method proposed here, the results are compared with those obtained using the FE method. The FE analysis is carried out using COMSOL Multiphysics® version 6.2 software [36]. Quadratic Lagrange rectangular elements with nine nodes are used, and a frequency domain analysis is performed using eight elements per shear wavelength for the highest frequency. The electrical impedances and mode shapes in terms of the total displacement of a double-layer piezoelectric stack are calculated and compared to the FE results first. Then, the effect of the number of layers on the electrical impedance is investigated.

### 4.1. Double-Layer Piezoelectric Stack

We consider a double-layer piezoelectric stack, as shown in Figure 2, consisting of two soft PZ27 (Ferroperm Piezoceramics [37]) disks with an electric voltage applied between the top and bottom faces of the stack (line-1 and line-5 in Figure 2a). Each piezoelectric disk has a density *ρ* of 7800 kg/m^3^, a radius *a* of 8 mm, and a thickness 2*b* of 1.13 mm. The material properties are provided by the manufacturer and are listed in *d*-form in Table 1. They are transformed into the *e*-form for our calculations, according to [38]. The two piezoelectric disks are connected in series-type and parallel-type configurations.

#### 4.1.1. Series-Type Configuration

**Figure 3:**
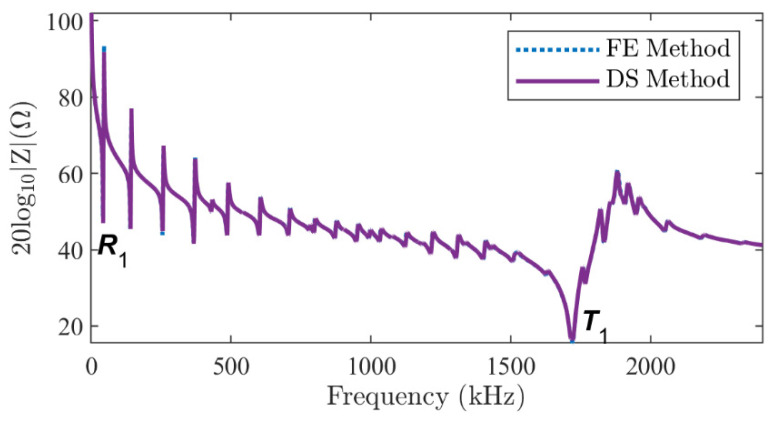
The modulus of the electrical impedance of a two-layer piezoelectric stack in a series-type configuration obtained by the FE method (dotted line) and the DS method proposed (solid line).

For this configuration, the electric potentials in Equation (25) are set to *φ*_1_ = 1 V and *φ*_2_ = 0 V, and the two piezoelectric elements have opposite poling directions. A frequency domain analysis is performed between 0 Hz and 2400 kHz with a step of 1 kHz. Figure 3 shows the modulus of electrical impedance using the *M* = 20 and *N* = 2 terms, with the FE results for comparison. The two sets of results overlap perfectly, with a correlation coefficient higher than 99.75%. The mean absolute percentage deviation (MAPD [21]) is 1.67%. In addition, the calculation time is reduced from the FE method’s 251.3 s to the DS method’s 8.19 s using Intel (R) Core (TM) i7-11800H CPU, 2.3 GHz with 32 GB memory at the same level of accuracy.

**Figure 4:**
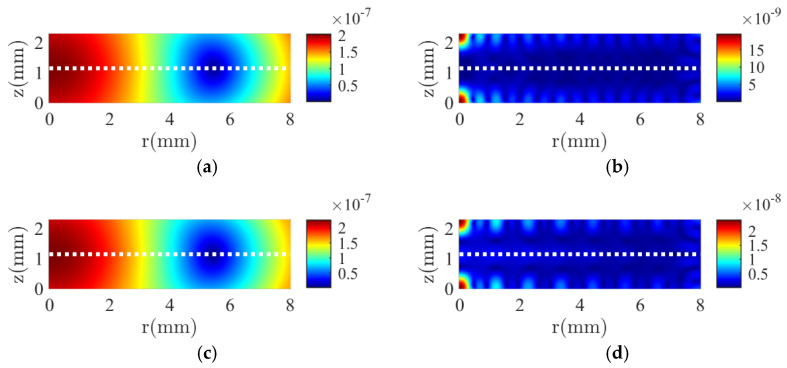
The total displacement of the two-layer piezoelectric stack in a series-type configuration at the resonance frequency of the first radial (*R*_1_) mode (**a**,**c**) and the first thickness (*T*_1_) mode (**b**,**d**). (**a**,**b**) indicate the results of the FE method; (**c**,**d**) indicate the results of the DS method.

The mode shapes of the total displacement $\sqrt{u^{2}+w^{2}}$ at the resonance frequencies of the *R*_1_ and *T*_1_ modes, as labeled in Figure 3, are shown in Figure 4. An excellent agreement is observed between the results of the DS method (Figure 4c,d) and the FE method (Figure 4a,b). Not only the spatial distribution along the *r* and *z* axes, but also the amplitude on the cross-section is almost identical. The vibration pattern of the *R*_1_ mode can be easily recognized from the left side of Figure 4: the piezoelectric stack extends mainly in the radial direction. It suffers little interference from the thickness mode since it lies in the low-frequency range. However, the vibration of the *T*_1_ mode is significantly influenced by the higher-order harmonics of the radial modes, as shown on the right side of Figure 4. Although the overall mode shape shows a vibration pattern in the *z* direction, the amplitude is modulated in the *r* direction by higher-order harmonics. In addition, the continuity boundary conditions along line-3 in Figure 2a (white dotted line) are satisfied.

**Figure 5:**
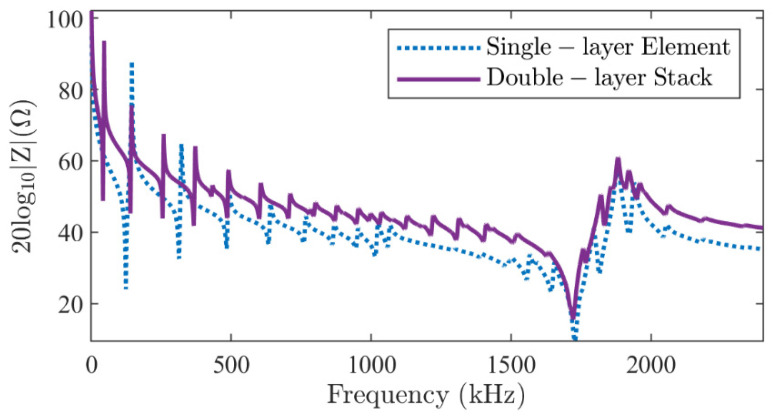
The modulus of the electrical impedance of a single-layer piezoelectric element (dotted line) and a two-layer piezoelectric stack in a series-type configuration (solid line) obtained from the DS method proposed.

Figure 5 shows the comparison of the electrical impedance of single- and double-layer stacks. Due to the coupling that comes with the addition of an element, the vibration pattern greatly changed. For the same voltage applied, the overall electrical impedance is doubled. Furthermore, additional vibration modes have emerged, such as a radial mode at 42 kHz. The number of resonance modes is increased from 27 to 35 in the frequency range from 0 to 2400 kHz.

#### 4.1.2. Parallel-Type Configuration

**Figure 6:**
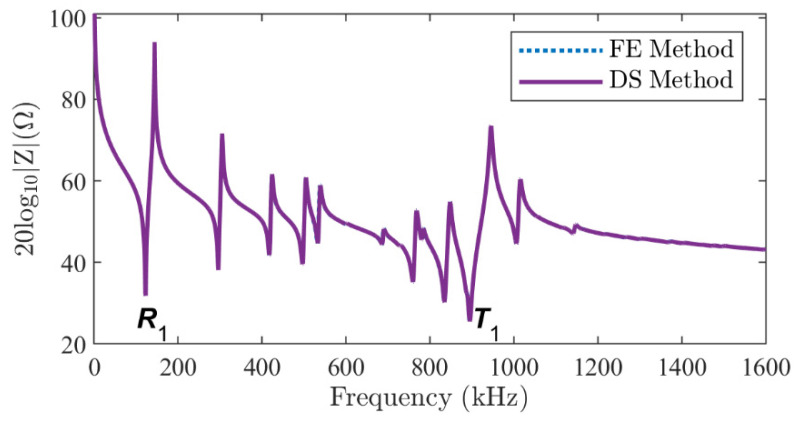
The modulus of the electrical impedance of a two-layer piezoelectric stack in a parallel-type configuration obtained by the FE method (dotted line) and DS method proposed (solid line).

In this situation, the two piezoelectric elements have the same poling directions. As shown in Figure 6, the two sets of results for the DS and FE methods overlap completely with a correlation coefficient higher than 99.99% and MAPD equal to 0.35%. The calculation time is reduced from the FE method’s 235.12 s to the DS method’s 10.06 s.

**Figure 7:**
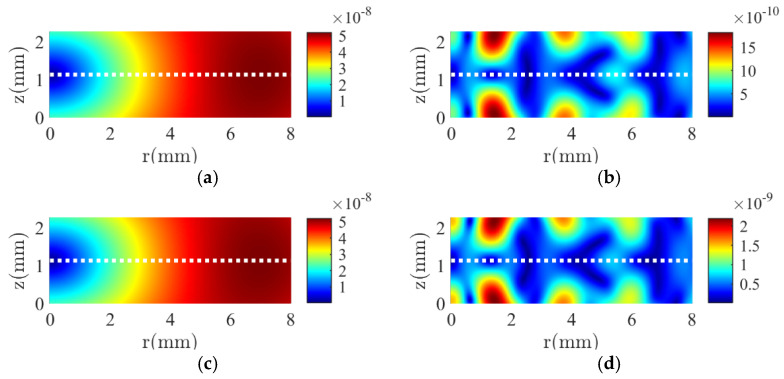
The total displacement of the two-layer piezoelectric stack in a parallel-type configuration at the resonance frequency of the first radial (*R*_1_) mode (**a**,**c**) and the first thickness (*T*_1_) mode (**b**,**d**). (**a**,**b**) indicate the results of the FE method; (**c**,**d**) indicate the results of the DS method.

The mode shapes at the resonance frequencies of the *R*_1_ and *T*_1_ modes are shown in Figure 7. The results of the two methods also agree very well, not only in the spatial distribution but also in the amplitude. The piezoelectric stack extends mainly in the radial direction in *R*_1_ mode. The maximum displacement occurs at the edge *r* = *a*, while it occurs at the center *r* = 0 in a series-type configuration (Figure 4a). The vibration of the thickness mode in the *z*-direction is more pronounced owing to the decrease of the resonance frequency from 1721 kHz to 895 kHz, which leads to a decrease in the radial mode order. The continuity boundary conditions along line-3 in Figure 2a (white dotted line) are also satisfied.

**Figure 8:**
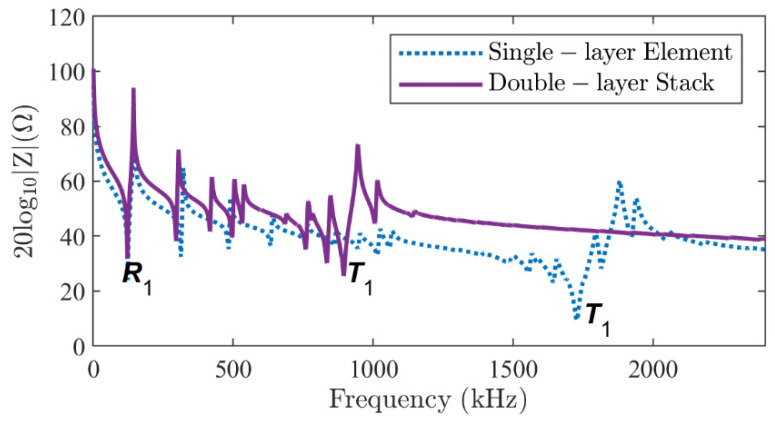
The modulus of the electrical impedance of a single-layer piezoelectric element (dotted line) and a two-layer piezoelectric stack in parallel-type configuration (solid line) obtained from the DS method proposed.

Figure 8 shows the comparison of the electrical impedance of single- and double-layer stacks. For the same voltage applied, the overall electrical impedance is also doubled. Due to the coupling between the two elements, the resonance frequency of the *R*_1_ mode slightly reduced from 124 kHz to 123 kHz (−0.81%), while the resonance frequency of the *T*_1_ mode greatly decreased from 1728 kHz to 895 kHz (−48.21%). In fact, the electrical impedance of a double-layer stack is equivalent to that of a single-layer double-thickness element. Due to the increased thickness, the resonance frequency of the *T*_1_ mode decreased by almost half.

### 4.2. Effect of Number of Layers

**Figure 9:**
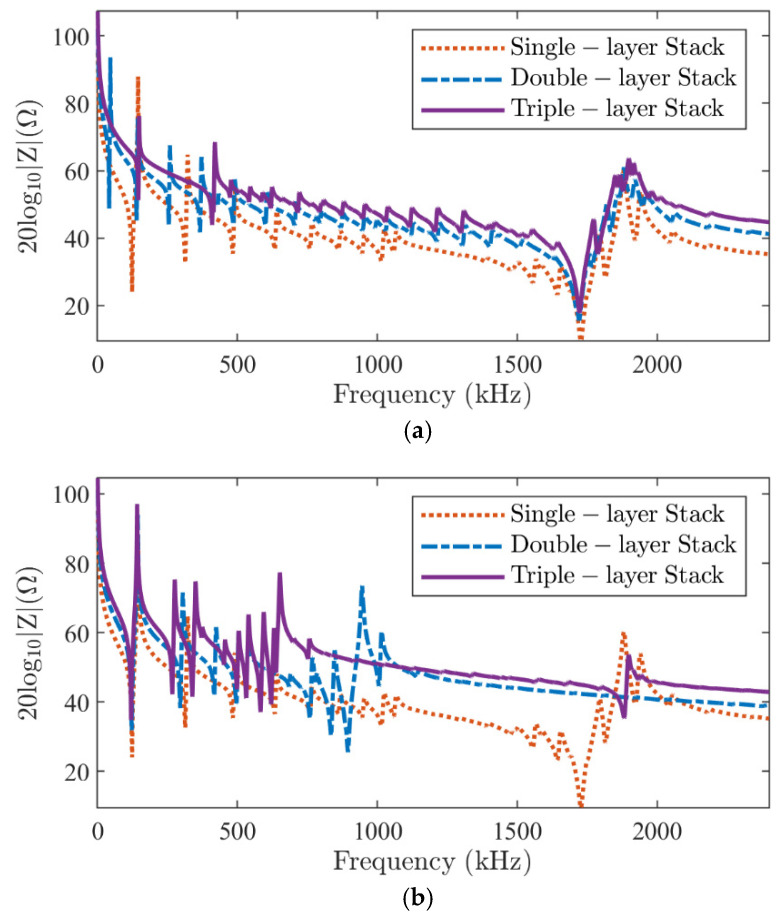
The modulus of the electrical impedance of a single-layer (dotted line), a two-layer (dash line), and a three-layer (solid line) piezoelectric stack in the series-type (**a**) and parallel-type (**b**) configuration obtained from the DS method proposed.

To further investigate the impact of the number of layers on electrical impedance, triple-layer stacks in series- and parallel-type configurations are studied. The dimensions of each layer are thickness 2*b* = 1.13 mm and radius *a* = 8 mm. The same soft PZ27 material is used. Similarly, when voltages of 0 V and 1 V are applied to the top and bottom surfaces, Figure 9 shows the results of a triple-layer stack with single- and double-layer results for comparison. Compared to the single-layer element, the electrical impedance is tripled.

In the series-type configuration (Figure 9a), the resonance frequency of the *T*_1_ mode does not change much; however, the antiresonance peak at around 1881 kHz is much more disturbed. The lowest radial peak of the double-layer stack at around 42 kHz disappears in the triple-layer stack. This may be due to the odd number of layers. In the parallel-type configuration (Figure 9b), the *T*_1_ mode again shifts to a lower frequency range, causing its third harmonics, *T*_3,_ to enter the frequency range of 0 to 2400 kHz. The electrical impedance indicates that the triple-layer stack is equivalent to that of a single-layer triple-thickness element.

## 5. Conclusions

In this work, a dynamic stiffness (DS) method for the modeling and characterization of multilayer piezoelectric stacks with the electric potential boundary conditions has been presented. The general solutions of the three vibration patterns—pure, symmetrically coupled, and anti-symmetrically coupled vibration modes—are derived first, and then they are superimposed to form the final solution. By virtue of the Projection method, the global DS matrix characterizing the relation between the generalized force and displacement is obtained. The dynamic responses and mode shapes of a piezoelectric stack consisting of two piezoelectric disks connected in series and in parallel are calculated. The comparison with the FE method shows excellent agreement, with a correlation coefficient higher than 99%. The calculation time has been reduced from hundreds of seconds to several seconds at the same level of accuracy. The method proposed here provides an efficient tool for the design and analysis of multilayer piezoelectric stack actuators.

## Figures and Tables

**Table 1:** Soft PZ27 piezoelectric material properties [37] *.

$s_{11}^{E}$	$s_{12}^{E}$	$s_{13}^{E}$	$s_{33}^{E}$	$s_{44}^{E}$	$d_{31}$	$d_{33}$	$d_{15}$	$\varepsilon_{11}^{T}$	$\varepsilon_{33}^{T}$	$\delta_{m}$	$\delta_{e}$
17	−6.6	−8.61	23.2	43.5	−17	42.5	50.6	1796	1803	1.35	1.7

* $s_{\alpha \beta }^{E}$ : elastic constants (×10^−12^ m^2^/N); $d_{i\alpha }$ : piezoelectric constants (×10^−9^ V/N); $\varepsilon_{ij}^{T}$ : relative dielectric constants; $\delta_{m}$ : mechanical loss factor (%); $\delta_{e}$ : dielectric loss factor (%).

## Data Availability

The original contributions presented in the study are included in the article, further inquiries can be directed to the corresponding author.

## Appendix A

First, let us express the components of symmetrically coupled vibration modes as

(A1) $$\begin{array}{l} u_{1}^{sc}=AJ_{1}\left(k_{me}r\right)\cos\left(k_{ne}z\right)\\ u_{3}^{sc}=BJ_{0}\left(k_{me}r\right)\sin\left(k_{ne}z\right)\\ \varphi^{sc}=CJ_{0}\left(k_{me}r\right)\sin\left(k_{ne}z\right) \end{array}$$

Substituting Equation (A1) into coupled field Equation (4), we obtain

(A2) $$\left[\begin{array}{ccc} -c_{11}^{E}k_{me}^{2}-c_{55}^{E}k_{ne}^{2}+{\rho \omega }^{2} & -\left(c_{13}^{E}+c_{55}^{E}\right)k_{me}k_{ne} & -\left(e_{31}+e_{15}\right)k_{me}k_{ne}\\ -\left(c_{13}^{E}+c_{55}^{E}\right)k_{me}k_{ne} & -c_{33}^{E}k_{ne}^{2}-c_{55}^{E}k_{me}^{2}+{\rho \omega }^{2} & -e_{15}k_{me}^{2}-e_{33}k_{ne}^{2}\\ -\left(e_{31}+e_{15}\right)k_{me}k_{ne} & -e_{15}k_{me}^{2}-e_{33}k_{ne}^{2} & \varepsilon_{11}^{S}k_{me}^{2}+\varepsilon_{33}^{S}k_{ne}^{2} \end{array}\right]\left[\begin{array}{l} A\\ B\\ C \end{array}\right]=0$$

This system of linear homogeneous equations in *A*, *B* and *C* yields nontrivial solutions when the determinant of the coefficient matrix vanishes. The determinant is quadratic in *ω*^2^ but cubic in $k_{me}^{2}$ and $k_{ne}^{2}$. Thus, for a given *ω* and *k_me_*, there are three different roots $k_{mei}^{2}$ (*i* =1,2,3), each of which generates an amplitude ratio when substituted in (A2). The amplitude ratio will be designated by

(A3) $$A_{mei}:B_{mei}:C_{mei}=1:\alpha_{1}^{mei}:\alpha_{2}^{mei}$$

Similarly, for a given *ω* and *k_ne_*, there are three different roots $k_{nei}^{2}$ (*i* =1,2,3). The amplitude ratio will be designated by

(A4) $$A_{nei}:B_{nei}:C_{nei}=1:\alpha_{1}^{nei}:\alpha_{2}^{nei}$$

The separated solutions of stresses and electric displacements in Equation (14) are

(A5) $$\begin{array}{cc} H_{3}\left(k_{mei}z\right)=\sum_{i=1}^{3}\alpha_{3}^{mei}A_{mei}\cos\left(k_{mei}z\right), & G_{3}\left(k_{nei}r\right)=\sum_{i=1}^{3}\alpha_{3}^{nei}A_{nei}J_{0}\left(k_{nei}r\right)\\ H_{4}\left(k_{mei}z\right)=\sum_{i=1}^{3}\alpha_{4}^{mei}A_{mei}\cos\left(k_{mei}z\right) & G_{4}\left(k_{nei}r\right)=\sum_{i=1}^{3}\alpha_{4}^{nei}A_{nei}J_{0}\left(k_{nei}r\right)\\ H_{5}\left(k_{mei}z\right)=\sum_{i=1}^{3}\alpha_{5}^{mei}A_{mei}\sin\left(k_{mei}z\right), & G_{5}\left(k_{nei}r\right)=\sum_{i=1}^{3}\alpha_{5}^{nei}A_{nei}J_{1}\left(k_{nei}r\right)\\ H_{6}\left(k_{mei}z\right)=\sum_{i=1}^{3}\alpha_{6}^{mei}A_{mei}\cos\left(k_{mei}z\right), & G_{6}\left(k_{nei}r\right)=\sum_{i=1}^{3}\alpha_{6}^{nei}A_{nei}J_{0}\left(k_{nei}r\right)\\ H_{7}\left(k_{mei}z\right)=\sum_{i=1}^{3}\alpha_{7}^{mei}A_{mei}\sin\left(k_{mei}z\right), & G_{7}\left(k_{nei}r\right)=\sum_{i=1}^{3}\alpha_{7}^{nei}A_{nei}J_{1}\left(k_{nei}r\right)\\ {\bar{H}}_{4}\left(k_{mei}z\right)=\sum_{i=1}^{+\infty }\gamma_{5}A_{mei}\cos\left(k_{mei}z\right), & {\bar{G}}_{4}\left(k_{nei}r\right)=\sum_{i=1}^{3}\frac{\gamma_{5}}{r}A_{nei}J_{1}\left(k_{nei}r\right) \end{array}$$

where

(A6) $$\begin{array}{cc} \alpha_{3}^{mei}=\left(c_{33}^{E}\alpha_{1}^{mei}+e_{33}\alpha_{2}^{mei}\right)k_{mei}+c_{13}^{E}k_{me}, & \alpha_{3}^{nei}=\left(c_{33}^{E}\alpha_{1}^{nei}+e_{33}\alpha_{2}^{nei}\right)k_{ne}+c_{13}^{E}k_{nei},\\ \alpha_{4}^{mei}=\left(c_{13}^{E}\alpha_{1}^{mei}+e_{31}\alpha_{2}^{mi}\right)k_{mei}+c_{11}^{E}k_{me}, & \alpha_{4}^{nei}=\left(c_{13}^{E}\alpha_{1}^{nei}+e_{31}\alpha_{2}^{nei}\right)k_{ne}+c_{11}^{E}k_{nei},\\ \alpha_{5}^{mei}=\left(-c_{55}^{E}\alpha_{1}^{mei}-e_{15}\alpha_{2}^{mei}\right)k_{me}-c_{55}^{E}k_{mei}, & \alpha_{5}^{nei}=\left(-c_{55}^{E}\alpha_{1}^{nei}-e_{15}\alpha_{2}^{nei}\right)k_{nei}-c_{55}^{E}k_{ne},\\ \alpha_{6}^{mei}=\left(e_{33}\alpha_{1}^{mei}-\varepsilon_{33}^{S}\alpha_{2}^{mei}\right)k_{mei}+e_{31}k_{me}, & \alpha_{6}^{nei}=\left(e_{33}\alpha_{1}^{nei}-\varepsilon_{33}^{S}\alpha_{2}^{nei}\right)k_{ne}+e_{31}k_{nei},\\ \alpha_{7}^{mei}=\left(-e_{15}\alpha_{1}^{mei}{+\varepsilon }_{11}^{S}\alpha_{2}^{mei}\right)k_{me}-e_{15}k_{mei}, & \alpha_{7}^{nei}=\left(-e_{15}\alpha_{1}^{nei}{+\varepsilon }_{11}^{S}\alpha_{2}^{nei}\right)k_{nei}-e_{15}k_{ne}. \end{array}$$
